# Virtual Network Embedding Based on Graph Entropy

**DOI:** 10.3390/e20050315

**Published:** 2018-04-25

**Authors:** Jingjing Zhang, Chenggui Zhao, Honggang Wu, Minghui Lin, Ren Duan

**Affiliations:** 1 School of Information, Yunnan University of Finance and Economics, Kunming 650221, China; 2 School of Continuing Education, Yunnan University of Finance and Economics, Kunming 650221, China

**Keywords:** graph entropy, virtual network embedding, probability, information measure, 68M10

## Abstract

For embedding virtual networks into a large scale substrate network, a massive amount of time is needed to search the resource space even if the scale of the virtual network is small. The complexity of searching the candidate resource will be reduced if candidates in substrate network can be located in a group of particularly matched areas, in which the resource distribution and communication structure of the substrate network exhibit a maximal similarity with the objective virtual network. This work proposes to discover the optimally suitable resource in a substrate network corresponding to the objective virtual network through comparison of their graph entropies. Aiming for this, the substrate network is divided into substructures referring to the importance of nodes in it, and the entropies of these substructures are calculated. The virtual network will be embedded preferentially into the substructure with the closest entropy if the substrate resource satisfies the demand of the virtual network. The experimental results validate that the efficiency of virtual network embedding can be improved through our proposal. Simultaneously, the quality of embedding has been guaranteed without significant degradation.

## 1. Introduction

### 1.1. Virtual Network Embedding

Network virtualization (NV) technology enables flexibility [1] on relatively rigid Internet architecture to accommodate gradually abundant Internet applications. Successful paradigms have emerged in cloud data centers for resource allocation [1], which stimulates a considerable volume of work for efficient solutions in this field. For realizing network virtualization, Internet Service Provider (ISP) must provide a mechanism for allocating substrate physical resources to provide user-expected services by ISPs. The resource allocation function in NV is presented as virtual network embedding (VNE), here “embedding” is also equivalently termed mapping, provisioning or assignment. Generally, VNE formulates user demand as virtual networks (VNs) consisting of virtual nodes connected by virtual links, and substrate physical resources as substrate network (SN).

Research on the VNE problem originated from finding the optimal solution in respect to some object to configure distributed substrate resource for fulfilling the user’s request. Theoretically, this requires mathematical optimization to formulate and solve the VNE problem accurately. Chowdhury et al. [2] extend the substrate network to an augmented substrate graph in which virtual nodes are connected to substrate nodes within some distance so as to merge both into a meta graph. Over the meta graph, the VNE problem is formulated by a mixed-integer program, and two approaches, deterministic D-ViNE and randomized R-ViNE, are devised for solving. Jarray et al. [3] decompose an overall VNE problem into a main part with constraints on the availability of substrate resource, and a pricing part with constraints on the embedding of VN requests, where competition among VN users is concerned through a periodical auction, and problem formulation is facilitated with column generation technology (CG). Besides parameters such as provider revenue, acceptance ratio, and embedding cost (see, e.g., [1]), Chen et al. [4] construct a minimization model of energy-efficient virtual node embedding and solve it to yield the minimal product of energy.

In parallel, heuristic solutions for the VNE problem receive much attention because researchers try to overcome the low implementation efficiency on mathematical optimization model. Yu et al. [5] advocate maximizing resource utilization in the substrate network. Hence the virtual nodes are greedily mapped to the substrate nodes with the maximum amount of substrate resources so as to minimize the use of the resources. Then virtual links are mapped to the shortest paths between two mapped substrate nodes. Lischka et al. [6] offer a VNE algorithm by detecting subgraph isomorphism between topologies of VN and SN to discover the correspondence between both nodes and links in the same stage. The VNE algorithm through isomorphism detection performs particularly efficiently, compared to other two-stage approaches on large VNs with high resource demands. Cheng et al. [7] propose a VNE strategy based on node ranking. Their approach ranks all virtual and substrate nodes according to their relative importance measured by the bandwidths on outgoing links, and the importance of all reachable nodes and out-neighbors. Then the higher-ranked virtual nodes have priority to be mapped to the higher-ranked substrate nodes. Virtual link mapping is implemented by the shortest-path algorithm or by the multi-commodity flow algorithm.

Recently, Beck et al. [8] design a distributed and parallel VNE framework called DPVNE, in which several VNE algorithms are executed in a distributive way, so that the single point pressure in SN is alleviated and greater efficiency is achieved. Zhang et al. [9] propose an opportunistic resource-sharing scheme to handle time-dependent virtual network requests (VNRs), in which the variable section is separated from required resources, and two solutions of allocating time slot are proposed such that bandwidth of virtual links can be mapped in corresponding time slots for realizing multiple VNs sharing substrate resources meanwhile.

Unfortunately, the VNE approaches introduced so far commonly encounter a challenge that they will consume unacceptable time when executing combinatorial search for solutions in large objective spaces. Naturally, we consider relying on graph entropy of the substructure for time complexity reduction. Such motivation arises from the fact that graph entropy can capture the randomness of the local substructure, which can confine the candidate objects of a demanded resource to a set of subnetworks having enough available resources with a high probability, because the magnitude and distribution of graph entropy substantially reflect the structural and geometric properties of the graph. There have been successful applications exploiting graph entropy to estimate the weights of indexes in system evaluation. This indicates that graph entropy is capable of capturing the random property of local graph structure so as to be able to guide the selection of candidate resources for VNE.

### 1.2. Graph Entropy Measures

Graph entropy measures have been extensively applied in a variety of problem areas with multiple forms of definition, such as discrete mathematics, computer science, information theory, statistics, finance, computational biology, knowledge mining, structural chemistry, etc. [10]. A majority of applications characterize networks through graph entropy measures to quantify the structural information content of these networks for capturing their complexity. There graph entropy was applied for measuring the network complexity in a probabilistic approach, differentiating with deterministic methods such as Kolmogorov complexity measure using encoding and substructure approach counting the number of the specified substructures (see [11] for details).

Generally, the Shannon entropy corresponding to Boltzmann entropy in thermodynamics has the following form:

(1) $$I_{S}=-\sum_{k}p_{k}\log(p_{k}),$$

which is a special form of the following Rényi entropy of order *q* in case of *q* = 1:

(2) $$I_{R}=\frac{1}{1-q}\log\left(\sum_{k}p_{k}^{q}\right).$$

For measuring topological information, classical graph entropy measures, originally defined and explored by Rashevsky [12], Trucco [13] and Mowshowitz [14], use intrinsic structural features of a graph to determine a probability distribution over the graph. Usually, a set *X* of graph elements, called the graph invariant that means the cardinality of *X* being invariant under graph isomorphisms, along with an equivalence relation *π* which induces a partition of *X* into *X_i_*, can define a probability distribution by letting *p_i_* = *P* (*v* ∈ *X_i_*) = |*X_i_*|/|*X*|. Based on this, the Shannon entropy formula *I_s_* can be applied to obtain a general definition of graph entropy as follows:

(3) $$I(G)=-\sum_{i=1}^{k}\frac{\left|X_{i}\right|}{\left|X\right|}\log\left(\frac{\left|X_{i}\right|}{\left|X\right|}\right).$$

Particularly, Rashevsky [12] defines *X* as the set of vertices, namely *X = V*(*G*), and *X_i_* as the *i*-th vertex orbit of *V*(*G*), where all orbits of *V*(*G*) are generated by the vertex automorphism group of *G*. Trucco [13] introduces similar entropy measures by setting *X* as the edge set of *G*, namely *X = E*(*G*), and *X_i_* as the *i*-th edge orbit under edge automorphism group. Mowshowitz [14] define *X* = *V*(*G*) and *X_i_* as *i*-th chromatic decomposition of the vertices.

Körner [15] introduces the first definition of graph entropy called Körner entropy, using an extrinsic probability distribution (not necessarily induced by graph invariant). Graph entropy measures based on the partition of graph elements are not computable for large networks. Recently, Dehmer [16,17] proposed the concept of parametric graph entropy, in which information functions of capturing structural features of a graph are designed to derive probability distribution on graph vertices, and graph entropy is measured by Shannon formula. In particular, the following information functions have been proposed [11]:

(4) $$f_{1}(v)=\alpha^{\sum_{k=1}^{\rho }c_{k}|s_{k}(v,G)|},\;\alpha >0,\;c_{k}>0,\;1\leq k\leq \rho (G),$$

where *ρ*(*G*) denotes the diameter of *G*;

(5) $$f_{2}(v)=\sum_{k=1}^{\rho (G)}c_{k}|S_{k}(v,G)|,\;\alpha >0,\;c_{k}>0,\;1\leq k\leq \rho (G);$$

(6) $$f_{3}(v)=|\lambda_{v}(G)|,$$

where *λ_v_* denotes the eigenvalue indexed by vertex *v* of the adjacency matrix *A*(*G*).

The graph entropies corresponding to these information functions can be uniformly defined by

(7) $$I_{f}(G)=-\sum_{i=1}^{\left|V\right|}\frac{f(v_{i})}{\sum_{j=1}^{\left|V\right|}f(v_{j})}\log\left(\frac{f(v_{i})}{\sum_{j=1}^{\left|V\right|}f(v_{j})}\right).$$

The research of applying information entropy to measure the structure information of networks was initiated in 1979, when Bonchev et al. [18] provided a complete index survey aiming to measure chemical molecules and atoms. Since then, information entropy theory has been applied to society network research to find the potentially interesting substructure of objective social networks [19,20]. Distinctly, we would like to apply graph entropy theory to communication networks whose nodes indicate communication end devices, and links represent communication lines (virtual or real), instead of nodes indicating persons or organizations and links representing relations between nodes. For our purpose, we are particularly interested in applying graph entropy for efficient virtual network embedding. Thus it is reasonable to consider using parametric graph entropy for quantifying a network, given that an information function is flexible enough to describe the properties of the node resources and link bandwidth.

## 2. Definition and Model of VNE

**Figure 1:**
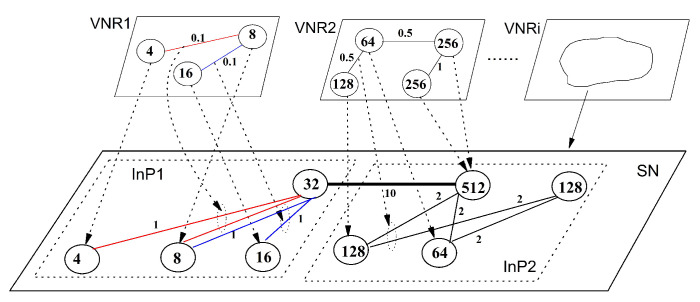
A two-level architectural model for virtual network embedding, with the correspondence between virtual edge and physical edge for InP2 omitted. The numbers in circles indicate the switching capacity of the routing and switching devices, and the ones near the links represent the transmission bandwidth, in Gbps. Virtual-to-physical edge correspondence is marked by distinct colors. VNR: virtual network request; SN: substrate network.

In network virtualization, the user demand is presented as a virtual network (VN). In VN, the nodes and links indicate the resource and communication demands, respectively. The virtual network embedding (VNE) algorithm, designed by a virtual network provider (VNP), embeds VN into SN by way of resource allocation. The VNE scheme imposes a substantial impact to the performance of the NV system. Thus, an efficient VNE solution pays a critical role in NV technology. Theoretically, VNE can be modeled as a generalized map from VN to SN, by which the graph *H* abstracting VN is embedded into graph *G* representing SN. The embedding must satisfy some constrains over the requested and provided resources, and it should optimize some parameters of interest to the user and virtual network provider (VNP), such as maximal provider revenue and accepted ratio, and minimal embedding cost [1]. To gain an intuition for grasping these notions, consider a two-level architectural model for virtual network embedding depicted in Figure 1, where a series of virtual network requests (VNR), presented as virtual networks, have been embedded into two substrate networks operated by two infrastructures InP1 and InP2.

Finding the optimal solution to a general graph embedding with constrains is an NP-hard problem. Much research has dealt with designing heuristic algorithms to solve it [5,6,7], an area which has received much attention in recent years with the spread of network virtualization.

Let graph *G* = (*V_G_*, *E_G_*) represent SN, where *V_G_* denotes the set of physical nodes and *E_G_* the set of physical links. Likewise, let *H* = (*V_H_*, *E_H_*) represent a virtual network request (VNR) from user, where *V_H_* denotes the set of virtual nodes and *E_H_* the set of virtual links. Let *c*(*x*) and *d*(*x*) be two functions representing the available resource and demand of network entity *x*, respectively. Then the problem of embedding *H* into *G* can be modeled as finding functions *f* which are subject to ∀*x* ∈ *G*, *d*(*x*) ≤ *c*(*f*(*x*)). If the objective is to minimize the cost of the embedding operation, the current known methods to find *f* can be characterized as solving the following optimization problem:

arg min*_f_* {*cost*(*f*) | *cost*(*f*)} = ∑*_e_*_∈_*_E_H__*∑*_l_*_∈_*_f_*_(*e*)_*cost*(*d*(*l*)) + ∑*_v_*_∈_*_V_H__*∑*_u_*_∈_*_f_*_(*v*)_*cost*(*d*(*u*)), (8)

where *cost*(*x*) denotes the cost of the variable *x*.

## 3. VNE Algorithm Using Graph Entropy

### 3.1. Algorithmic Profile

**Figure 2:**
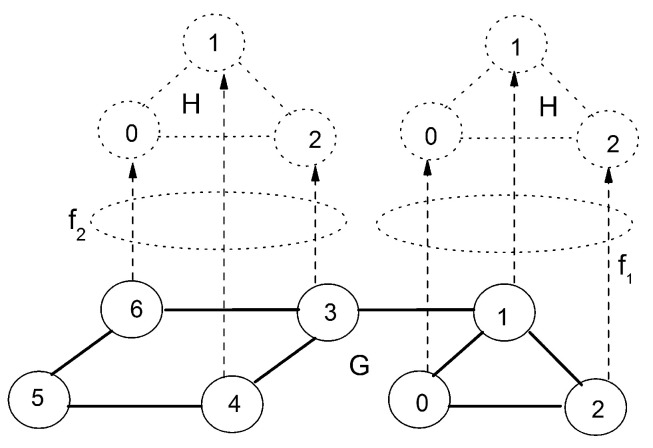
An example of embedding same one virtual network (VN) into two subnetworks.

For large substrate network *G*, the computational burden of searching resource will be relieved if candidates in substrate network can be confined in a particularly selected resource space, in which the distribution of resources exhibit a maximal similarity with the virtual network *H*. As an example, it can be observed in Figure 2 that embedding *f*_1_ demonstrates an apparent dominance relative to embedding *f*_2_ due to consideration of structural correspondence. The main idea of this work is to discover the structural similarity between substructures of substrate network *G* and the virtual network *H* through comparison of their graph entropies. Through graph entropy, the structure information of virtual and substrate networks can be quantified as respective entropies, which reflects the discrepancy of two compared structures, and narrows the space of objective solutions. More details regarding graph entropy measure can be found in [10,11,21].

The VNE algorithm based on graph entropy (GE-VNE) includes three procedures. In the first procedure, the algorithm searches a set of SN areas as candidates for VN embedding. These areas share common links or nodes. Then the algorithm detects one of the candidates with most resource as the optimal embedding area. In the second procedure, the algorithm searches all objects electable to fulfill the demands of VN in found candidates. These objects are structures simpler than those in the first procedure. In the final procedure, the graph entropies are calculated for all objects found in the second procedure, then the optimal candidate is found, and resource assignment will be implemented.

### 3.2. Selection of Candidate Areas

In the first stage of algorithm GE-VNE, for searching all SN substructures eligible to fulfill the virtual network request, procedures (described in Procedures 1 and 2) calculate the importance of all nodes. A natural way for completing this is to consider the quantity of node resource and the bandwidth of links incident to this node. Usually, SN nodes, holding more available resource and associating more available bandwidth, appear to be more important than those with less. Also, VN nodes are relatively important if they have high demands for resources. Consequently, the resource quantity of a node can indicate its importance (weights). Similarly, links transmitting heavy traffic mean large bandwidth demand in VN or resource in SN so that link bandwidth can characterize its weight. Because a virtual node can merely be embedded into a substrate node, the computation of graph entropy only involves in link attributes, regardless of node weights. 

Then algorithm GE-VNE selects areas centered at nodes with higher importance in order as candidate Areas. In order to apply graph entropy for embedding virtual network, we define the importance of a node in network *G* as the sum of resource magnitude *c*(*v*) plus the product of the number of links and the minimal bandwidth incident to *v*, namely,

(9) w(v)=c(v)+|E(v)|×min{c(l)|l∈E(v)},

where *E*(*v*) denotes the set of links incident to *v*, and *c*(*v*) denotes the available resource at entity *v*.

It seems to be reasonable that the best option area is the one with resource distribution closest to the VN. When multiple candidate areas are available for a customized virtual network request. For realizing this, it needs to estimate the resource distribution of all candidates in SN for objective VN. Let *S_i_* denote the *i*-th candidate of a VN, its quantity *a*(*S_i_*) of available resource can be defined as

(10) $$a(S_{i})=\sum_{l_{i}\in E(S_{i})}a(l_{i})+\sum_{u\in V(S_{i})}a(u).$$

By the above formula, the quantity of available resource in *S_i_* has been expressed as the sum of all available resource in nodes and links within candidate *S_i_*. Our algorithm would select *S_i_* with greatest value *a*(*S_i_*) as the candidate area. In order to confine the coverage of embedding *H* into *G*, let *ρ*(*G*) and *r*(*G*) denote the diameter and the radius of graph *G*, respectively. We define the radius *r*(*G*) of searching SN resource as follows:

(11) $$r(G)=w_{0}d_{0}+w_{1}|V(H)|+w_{2}|E(H)|,$$

where *d*_0_ denotes the average of all SN node pairwise distances, *w*_0_, *w*_1_, *w*_2_ assign initial distance, node and link weights, respectively.

### 3.3. Computation of VN and SN Entropies

Subsequently, the algorithm proceeds to choose the best suitable one for VN embedding among all candidates by measuring their graph entropies, as described in Procedure 3. For measuring the parametric graph entropies *I*(*H*) and *I*(*G*) proposed by Dehmer [16,17] (see Section 1.2 for details), it is required to define and calculate *f*(*v*) for all *v* ∈ *V*(*G*) firstly, where *f*(*v*) is a function quantifying the local structural information at each node *v*. It is particularly essential that defining *f*(*v*) to accurately quantize the structural information of graph *G*. There are a few of methods to define *f*(*v*) [10,11], depending on concrete application scenarios. The most common way is to define *f*(*v*) in terms of structural features around *v*, such as the node degree *δ*(*v*) and the number of paths across *v*. Apparently, a well-defined *f*(*v*) affects substantially the computation of graph entropy. As a challenge still remained, quantifying finely local information burdens computation overhead, and coarsely quantified one may weaken the ability of characterizing the objective graph.

To define *f*(*v*), denote substrate network with graph *G* defined as *G* = (*V*, *E*, *P*), in which *V* and *E* indicate the traditional sets of nodes and edges, respectively. Additionally, *P* is a probability function defined over graph *G*. For *f*(*v*) proposed by Dehmer in [16,17], it is necessary to know *S_k_*(*v*, *G*) in advance, where *S_k_*(*v*, *G*) represents a vertex subset of *V*(*G*) containing nodes in distance *k* from *v*, is called the *k*-sphere of *v* regarding *G*, namely,

*S_k_*(*v*, *G*) = {*u* ∈ *V*|*d*(*v*, *u*) ≤ *r_k_*, 1 ≤ *k*}. (12)

**Figure 3:**
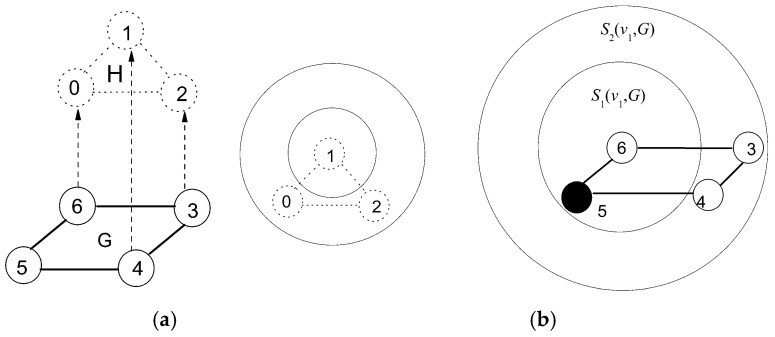
Entropy expression on structure information of VN and SN. (a) Structures of VN, SN, (b) Definitions of *S_k_*(*v*, *G*) and *S_k_*(*v*, *H*).

Defining *S_k_*(*v*, *G*) using large increment *k* hardly perceives the structure discrepancy. Reversely, a small increment *k* maybe leads to a high redundant computation. Generally, VN has relatively simpler structural attributes than SN in node and link distribution. Thus, *S_k_*(*v*, *G*) should be defined separately in VN and SN to avoid the problem described above. Additionally, the hidden node (marked as black solid circle) should be taken into account for graph entropy computation. As an example, Figure 3a shows that a VN of three-node circle will be embedded into a SN of four-node circle, and VN is divided as *S*_1_(*v*, *H*) and *S*_2_(*v*, *H*). In Figure 3b, SN is partitioned as *S*_1_(*v*, *H*) = {*v*_5_, *v*_6_} and *S*_2_ = (*v*_3_, *v*_4_). It is worth noting that candidate substructure for VN embedding contains a hidden node *v*_5_ which should be considered in process of computing *f*(*v*) to reflect the differences between candidates even if this hidden node is transparent to users.

Suppose that all values of bandwidth resources lie in interval [*a*, *b*] (units: Mb/s). We firstly divide [*a*, *b*] into *i* parts with same length. Then the radius *r_k_*(*G*) of sphere *S_k_*(*v*, *G*) can be set up as *r_k_*(*G*) = *a* + *k*(*b* − *a*)/*i*. The value of *r_k_*(*G*) can be adjusted by taking different values of the parameter *i* for VN and SN. The radius of *S_k_*(*v*, *H*) can be assigned by a same approach, instead of parameters *a* and *b* representing demands rather than resources. For a weighted graph *G*, the distribution of node weights are tightly connected to the values of |*S_k_*(*v*, *G*)|. If a node *u* is incident to links with high bandwidth in *S_k_*(*v*, *G*), it should contribute more to the whole network communication function, namely, the probability distribution *P* over *G* has a high value *P*(*u*) at node *u*. Generally, the weight coefficients *c_k_* of *S_k_*(*v*, *G*) are arranged as an increasing arithmetic series to express the relation between link bandwidth incident to node *u* and probability distribution *P*.

Then, the local information function *f*(*v*) on vertex set *V* can be defined as

(13) $$f(v)=\alpha^{\sum_{k=1}^{\rho }c_{k}|s_{k}(v,G)|},$$

where *α* nd *c_k_* are arbitrary and real positive coefficients, and |*S_k_*(*v*, *G*)| indicates the number of nodes located in interior of sphere centered at node *v* with radius *k*. Generally, one takes constants *c_k_* (1 ≤ *k* ≤ *ρ*) as an arithmetic sequence of positive integers. Letting $\rho_{s}(v)=\underset{1\leq k\leq \rho }{\max}|S_{k}(v,G)|$, $\rho_{c}=\underset{1\leq k\leq \rho }{\max}c_{k}$ and $r_{c}=\underset{1\leq k\leq \rho }{\min}c_{k}$ to guarantee the graph entropy *I_f_*(*G*) derived from Formula (5) being bounded, the parameter *α* should satisfy inequality (see [17]):

(14) $$1<\alpha \leq |V{|}^{\frac{1}{\rho [\rho_{s}(v)\rho_{c}-r_{c}]}}.$$

**Figure 4:**
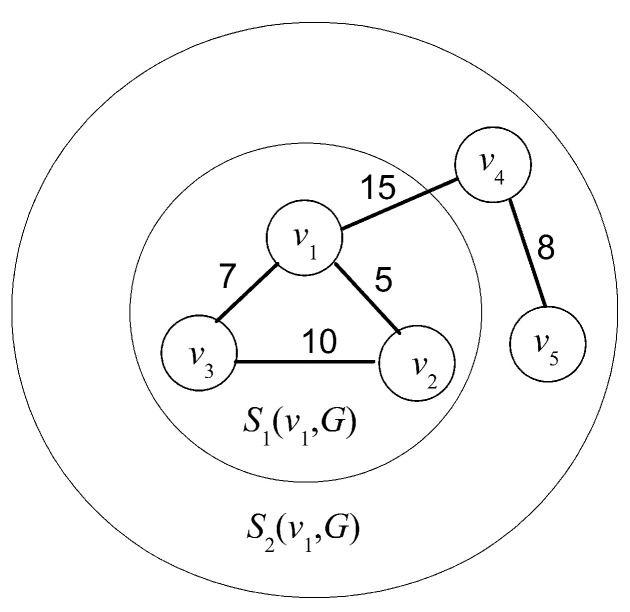
Computation of *S_k_*(*v*, *G*) in a five-node example network.

To understand the process of graph entropy, Figure 4 instantiates a five-node network centered at node *v*_1_, where all bandwidth resource lies in interval [10, 20]. Setting *i =* 2 it yields that *r*_1_(*G*) = 10 and *r*_2_(*G*) = 20 thus *S*_1_(*v*, *G*) and *S*_2_(*v*, *G*) are located at areas marked by large and small profiles of sphere, respectively. Further Setting up *c_k_* = *k*, it yields *S*_1_(*v*_1_, *G*) = {*v*_2_, *v*_3_} and *S*_2_(*v*_1_, *G*) = {*v*_4_} (|*S*_1_(*v*_1_, *G*)| = 2 and |*S*_1_(*v*_1_, *G*)| = 1). It follows from observation that *ρ*(*G*) = 3, *ρ_s_*(*v*_1_) = 2, *ρ_c_* = 2, *r_c_* = 1 and *ρ*[*ρ_s_*(*v*_1_)*ρ_c_* − *r_c_*] = 9. Thus the parameter *α* is bounded at (1, 5^1/9^] by Formula (14). Taking *α* = 5^1/10^ ∈ (1, 5^1/9^], it yields *f*(*v*) = 5^1/10(1 × 2 + 2 × 1)^ = 5^0.4^.

Once *f*(*v*) is known, the function value *p_i_* = *P*(*v_i_*) on vertex *v_i_* of probability distribution *P* can be computed by formula:

(15) $$p_{i}=\frac{f(v_{i})}{\sum_{v_{i}\in V(G)}f(v_{i})},$$

Then the entropy *I*(*G*) of graph *G* derives from the following expression in terms of probability distribution of *G*:

(16) $$I(G)=-\sum_{v_{i}\in V(G)}p_{i}\log(p_{i}),$$

### 3.4. Presentation of Algorithmic Pseudocodes

Based on definitions and formulas presented above, we commence to describe the details of our algorithm. The relevant procedures may be further described as algorithmic pseudocodes, with corresponding step-by-step comments, listed as follows.
**Algorithm 1. GE-VNE (Procedure 1):** Embedding Areas SearchInput: SN, VNOutput: {*S_i_*}1. for each node *u_i_*(1 ≤ *i* ≤ *n*) in SN2. calculate *w*(*u_i_*);3. end for4. sort {*u_i_*|1 ≤ *i* ≤ *n*} as {*μ_i_*|1 ≤ *i* ≤ *n*} by *w*(*u_i_*) in descending order;5. select *p* top important nodes {*μ_i_*|1 ≤ *i* ≤ *p*};6. for each *μ_i_* ∈ {*μ_i_*|1 ≤ *i* ≤ *p*}7. *S_i_*←*S_k_*(*μ_i_*, *G*, *r_k_*(*G*));8. end for9. *S_c_*←*S*_1_;10. for each *S_i_* in {*S_i_*|1 ≤ *i* ≤ *p*}11. calculate *a*(*S_i_*);12. if *a*(*S_i_*) > *a*(*S_c_*)13.  *S_c_*←*S_i_*;14. end if15. end for16. output *S_c_* as the candidate area for VN embedding.

In Procedure 1 (Algorithm 1):

Lines 1–3: calculate the importance of each node in SN by Formula (9);

Lines 4–5: select *p* top nodes {*μ_i_*|1 ≤ *i* ≤ *p*} according their importance;

Lines 6–8: for each of nodes {*μ_i_*|1 ≤ *i* ≤ *p*}, calculate a network area centered in *μ_i_* with radius *r*, as candidate areas for VN embedding, where *r* can be computed by Formula (11). Consequently, the embedding candidates for VN should have *p* areas: *S*_1_–*S_p_*.

Line 9–15: calculate the quantity *a*(*S_i_*) for each network area *S_i_*, and select the largest one *S_c_*.

Line12: output the selected embedding area *S_c_*;

**Algorithm 2. GE-VNE (Procedure 2):** Embedding Candidates Searching1. sort all virtual nodes by their importance as *V*(*H*) = {*v*_1_, *v*_2_, …, *v_n_*};2. sort all substrate nodes by their importance as *S* = {*u*_1_, *u*_2_, …, *u_n_*};3. initialize the candidate of VN as *S*_11_ = null4. for each node *v_i_* in *V*(*H*) 5. search top *s* nodes of SN which fulfill the demand of node *v_i_*: *T_i_* = {*u_i_|*1 ≤ *i* ≤ *s*, *u_i_* ∈ *V*(*G*), *c*(*u_i_*) ≥ *d*(*v_i_*)}6. for each node *u_j_* in *T_i_* *S_ij_* = *S*_(*i*−1)j_∪*u_j_*7. end for8. end for;9. output *S_ij_*

In Procedure 2 (Algorithm 2), as the central part of algorithm GE-VNE, search all SN substructures eligible to fulfill the virtual network request. Here introduce this procedure firstly, then an example is provided to facilitate understanding the implementation of GE-VNE.

**Figure 5:**
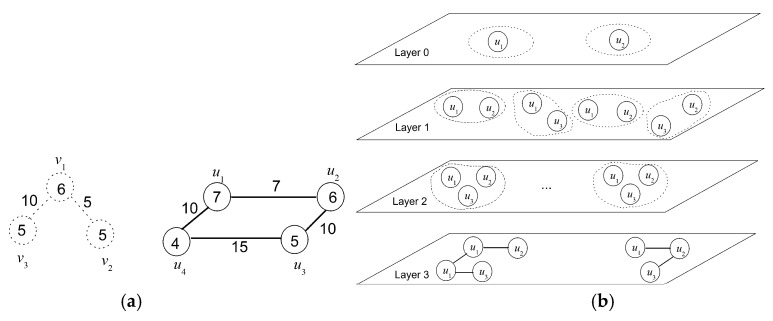
An example of finding a candidate of VN in SN, (**a**) VN (**left**) and SN (**right**), (**b**) searching candidates in SN.

In Figure 5, the VN node with largest importance is *v*_1_, and all candidates for *v*_1_ are {*u*_1_, *u*_2_, *u*_3_}. If setting *s* = 2, it is readily observed that *T*_1_ = {*u*_1_, *u*_2_}, *T*_2_ = *T*_3_ = {*u*_1_, *u*_2_, *u*_3_}, and *S*_1_ = {*u*_1_}, *S*_2_ = {*u*_2_}; *S*_11_ = {*u*_1_, *u*_1_}, *S*_12_ = {*u*_2_, *u*_1_}, *S*_21_ = {*u*_1_, *u*_2_}, *S*_22_ = {*u*_2_, *u*_2_}, *S*_13_ = {*u*_1_, *u*_3_}, *S*_23_ = {*u*_2_, *u*_3_}; *S*_111_ = {*u*_1_, *u*_1_, *u*_1_}, *S*_211_ = {*u*_2_, *u*_1_, *u*_1_}, *S*_121_ = {*u*_1_, *u*_2_, *u*_1_}, *S*_221_ = {*u*_2_, *u*_2_, *u*_1_}, *S*_131_ = {*u*_1_, *u*_3_, *u*_1_}, *S*_231_ = {*u*_2_, *u*_3_, *u*_1_}; notably, the elements in sets here are assumed to be in order for corresponding to the ordered set {*v*_1_, *v*_2_, …, *v_n_*} of virtual nodes, which makes {*u*_3_, *u*_1_} and {*u*_1_, *u*_3_} be different sets. Once procedure proceeds to its end, it will yield all expected candidates for objective VN.

**Algorithm 3. GE-VNE (Procedure 3):** VNE based on graph entropyInput: {*S_c_*_1_, *S_c_*_1_, …, *S_cr_*}, *H*Output: *f*: *H*→*G*1. calculate entropy *I*(*H*) of *H*2. for each *S_ci_* in {*S_c_*_1_, *S_c_*_2_, …, *S_cr_*}3. calculate entropy *I*(*S_ci_*);4. *d*(*I*(*S_ci_*))←|*I*(*S_ci_*) − *I*(*H*)|5. end for6. sort {*S_c_*_1_, *S_c_*_2_, …, *S_cr_*} as {*T_c_*_1_, *T_c_*_2_, …, *T_cr_*} by *d*(*I*(*S_ci_*)) as ascending order7. for each *T_ci_* in {*T_c_*_1_, *T_c_*_2_, …, *T_cr_*}8. if (*T_ci_* fulfills VN)9.   break;10. end if;11. end for 

In Procedure 3 (Algorithm 3), algorithm GE-VNE chooses the best suitable one for VN among all candidates by measuring their graph entropies, as described in details listed as follows.

Line 1: calculate the entropy *I*(*H*) of graph *H*;

Line 2–5: calculate the entropies *I*(*S_ci_*) of graph series *S_ci_*, and the entropy distances |*I*(*S_ci_*) − *I*(*H*)| between graphs *S_ci_* and *H*;

Line 6: sort *S_ci_* by entropy distance in ascending order;

Line 7–9: travel *S_ci_* in order until finding one which fulfills the demand of *H*.

### 3.5. Limitations of This Work

**Figure 6:**
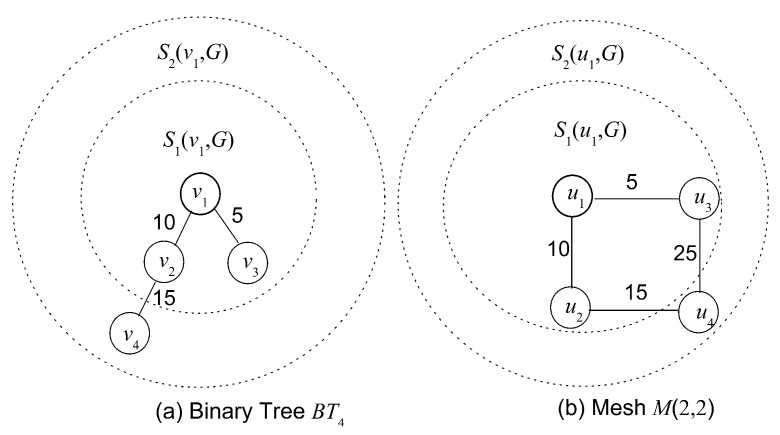
An example of exhibiting probability of equal entropies existing in two different topologies: (**a**) Binary Tree *BT*_4_ and (**b**) 2 × 2 Mesh *M*(2, 2).

The major limitation of algorithm GE-VNE is that entropy measures capture the structural attributes of different graphs efficiently, for example, the degree distribution. However, values from entropy measures are evidently inadequate in reflecting the topological discrepancy between graphs, particularly in cases of characterizing topological similarities on small graphs. The reason behind this can be understood readily from the definition of *f*(*v*) in Formula (13), where two arbitrary nodes *u* and *v* yield identical functional value *f*(·) as long as *S_k_*(*u*, *G*) = *S_k_*(*v*, *G*). This coincidence will happen in a high probability when the scales of objective networks are small. For example, Figure 6 exposes that two different graphs, (a) the Binary Tree *BT*_4_ and (b) 2 × 2 Mesh *M*(2, 2), might have the same entropy value though they are distinguished topologically, based on an observation that it holds *S_j_*(*v_i_*, *BT*_4_) = *S_j_*(*u*, *M*(2, 2)) (1 ≤ *j* ≤ *k*, 1 ≤ *i* ≤ 4)) for all nodes by setting same parameters *α*, *c_k_*, *r*_1_(*G*) = 10, *r*_2_(*G*) = 20 and *k* = 2 on both graphs. Given that two adjacent virtual nodes may be mapped to two separate substrate nodes connected by a path, the optimization for the VNE problem sometimes advocates of charactering graphs through structural attributes rather than contrasting their topological similarity.

## 4. Experiments

### 4.1. Experimental Configuration

In this section, we report on the results in a number of simulations conducted to experimentally validate performance and quality of algorithm GE-VNE. The experimental platform is facilitated with software IDE Eclipse (Neon, Eclipse Foundation, Ottawa, ON, Canada) under the 32-bit Windows 7 operating system (Microsoft Corporation, Redmond, WA, USA), and hardware CPU Intel(R) Core(TM) i7 5600-U @2.6 GHz with 8.0 GB RAM. All simulations generate the results with Alevin 2.1, developed by Beck et al. [22], that has successfully functioned as a simulation framework for examining virtual network embedding algorithms.

We encode the algorithm with programming language Java to generate subclass extending the class GenericMappingAlgorithm that has been realized as an algorithmic framework of generic VNE algorithms, and implemented all simulations under various scenarios. The experimental process consists of network generation, algorithm configuration and execution, and algorithm evaluation, with various experimental configurations.

The experimental steps and corresponding configurations are further detailed in Table 1 and Table 2. Also, a comparison with a couple of representative VNE algorithms, that have been cited as the focus of considerable VNE research, has been conducted in terms of runtime, VNR acceptance ratio, cost revenue ratio (cost/revenue), and node utilization ratio, such factors that have been recognized as effective factors of assessing VNE algorithms. Eventually, the results of comparison have been figured to perceive the performance and quality of algorithms in comparison.

• *Scenario Generation*

The theoretical analysis in the previous section focuses on using graph attributes for charactering graphs rather than topological association. This reminds us to conduct simulations paying less attention to the variety of network topologies. Thus two network topologies, the Binary Tree with 100 nodes (*BT*_100_), and the 7 × 7 Mesh *M*(2, 2), are selected as the SN models. Both networks have been recognized as practical topologies modeling datacenters in clouding computing environment and the Internet, also emerged as representative architectures for high performance computing, and appears to be cumulatively essential in era of undergoing multicore computer [23]. The step of network generation goes through establishing a network topology, adding resources to SN, as well as adding demands to VN. Additionally, VN topologies are randomly generated as 20 networks with sizes from 1 to 4. All SN node and bandwidth resources are randomly generated in interval [50, 100], and VN node and bandwidth demands are randomly generated in intervals [1, 20] and [1, 50], respectively (Units: Mb/s).

• *Algorithm Configuration*

Algorithms chosen for experimental evaluation involve five representative VNE algorithms, which have been proposed in [1,4,6] described in Table 3. These algorithms along with GE-VNE are executed under identical scenarios and parameter configurations. Evaluations are run 40–50 times in order to observe the performance of all algorithms. In the stage of mapping nodes, the weights of CPU nodes are set to 1, and the candidates of a VN node are limited within a distance of 20 hops away from it. The situation of node overload has not yet been considered. In the stage of mapping links, the parameter *k* of mapping a VN link to a length-*k* shortest path is set to *k* = 2. The other parameters pertaining to our algorithm are listed in Table 2.

### 4.2. Experimental Results

In order to evaluate performance of the algorithm GE-VNE, six metrics with respect to performance are considered in our experiment: average links stress (ALS); VNR acceptance ratio (AR); cost/revenue ratio (CRR); cost; link utilization (LU); and link cost per VNR (LCPV). The acceptance ratio reflects the fraction of VNRs successfully embedded as virtual networks. The revenue sums the revenue of the VNRs that were successfully mapped and the revenue of those that were not mapped. The cost measures the quantity of substrate resources allocated for VNR. The link utilization reflects the proportion of bandwidth being utilized to meet the currently accepted VNRs. The concrete implications of these VNE evaluation metrics have been interpreted in Table 4. Three conclusions can be observed from simulations.

**Figure 7:**
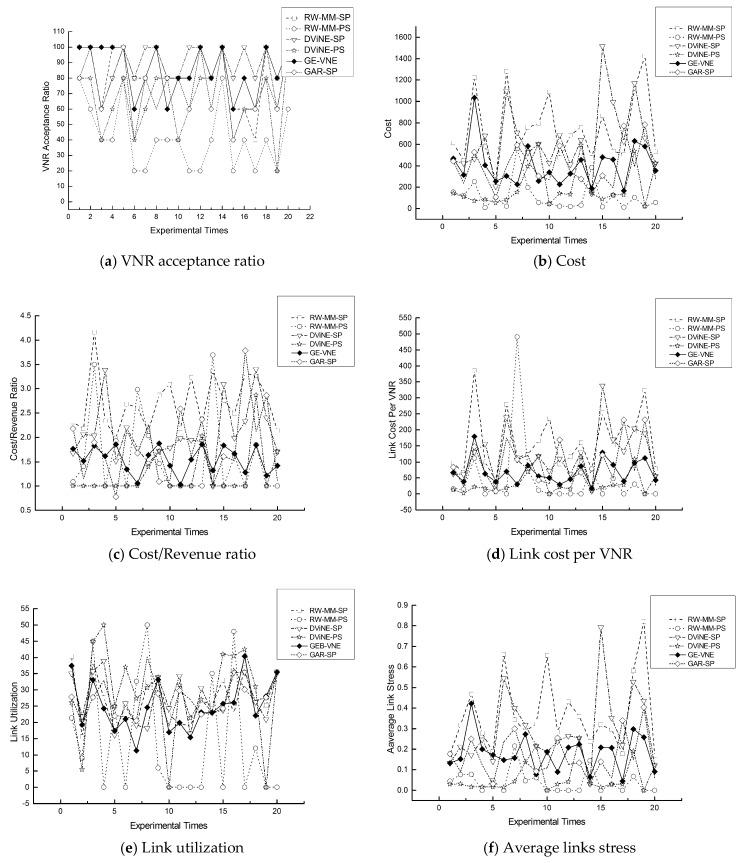
The comparisons of GE-VNE with other representative VNE approaches in terms of six VNE metrics, (**a**) AR; (**b**) Cost; (**c**) CRR; (**d**) LCPV; (**e**) LU; and (**f**) ALS, from executing algorithms 20 times on 7 × 7 Mesh *M*(7, 7).

**Figure 8:**
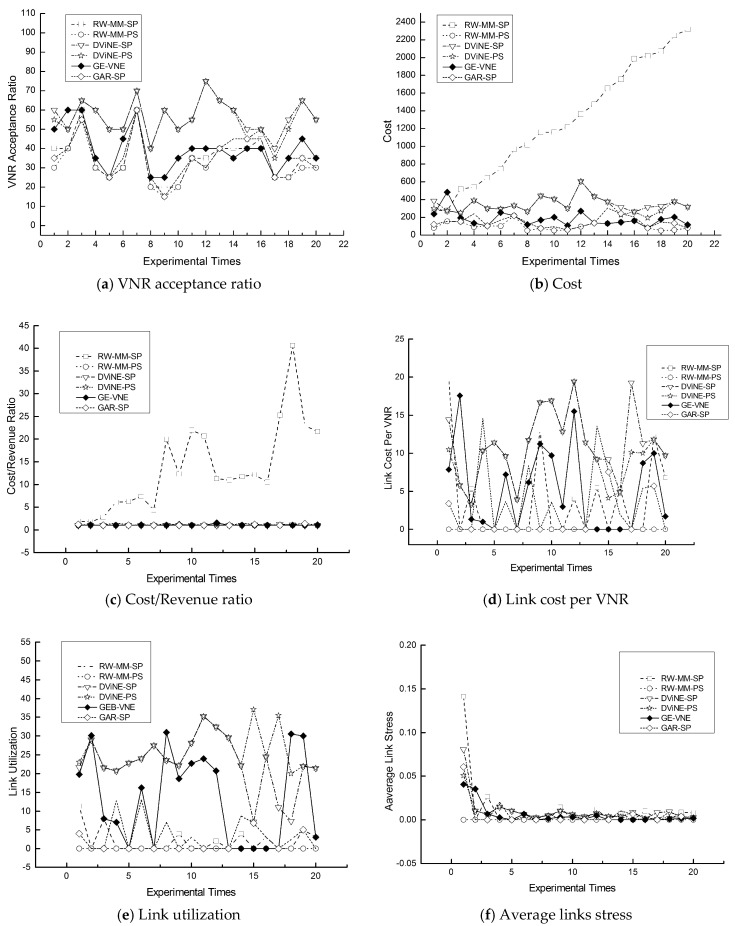
The comparisons of GE-VNE with other representative VNE approaches in terms of six VNE metrics, (**a**) AR; (**b**) Cost; (**c**) CRR; (**d**) LCPV; (**e**) LU; and (**f**) ALS, from executing algorithms 20 times on *BT*_100_.

In respect to the VNR acceptance ratio, Figure 7a displays that GE-VNE behaves comparatively better to other algorithms; also observed in Table 5, the VNR acceptance ratio caused by GE-VNE increases 41.25% and 59% to DViNE-SP and DViNE-PS, respectively. In Figure 8a, GE-VNE leads to a moderate VNR acceptance ratio as executing it on substrate network *BT*_100_.

Regarding three cost-related metrics: cost, cost/revenue, and average link cost, Figure 7 and Figure 8b–d indicate that algorithm GE-VNE yields relative low values to other algorithms as implementing them on *M*(7, 7) and *BT*_100_ for 20 times, also seen in Table 5. An extra should be observed in mentioned figures that GE-VNE holds a higher value than RW-MM-PS and DViNE-PS in average link cost, but latter two algorithms lead to a pretty low acceptance ratio 15% and 40.75%, respectively. Only algorithm DViNE-PS approaches GE-VNE in VNR acceptance ratio, but the former spends link cost 154.61, considerably higher than 84.83 by GE-VNE. Therefore, GE-VNE reduces the link cost in the situation of increasing VNR acceptance ratio. A promotion in whole embedding cost emerging in Figure 7b, is attributed to higher cost for node embedding for higher VNR acceptance ratio.

Figure 7 and Figure 8e,f illustrate improvements in link utilization and the average stress for 20 times VN embedding on experimental scenarios of embedding a random VN into 7 × 7 Mesh *M*(7, 7) in Figure 7 and *BT*_100_ in Figure 8. By definition, high link cost or VNR acceptance causes link stress increasing, which emerges in Figure 7f because of appearing a high VNR acceptance in GE-VNE, also seen in Table 5. As depicted in both Figure 7 and Figure 8, GE-VNE earns a better trade-off in VNR acceptance and link stress than DViNE-SP, and overall, reduces the average stress of each embedded VN to the whole substrate network on both *M*(7, 7) and *BT*_100_.

## 5. Conclusions

The particularly essential part for virtual network embedding is to choose an optimal subnetwork as candidate of the virtual network to be embedded. Previous VNE algorithms mainly concern the optimization of node and link embedding relative to some anticipated parameters, neglecting the impact of substrate network structure on VNE.

We propose a VNE algorithm based on graph entropy called GE-VNE to extract and quantify the structure information for both VN and SN. GE-VNE divides SN into substructures, and considers multiple available candidate structures for embedding objective VN, then searches all resources on SN to find the richest one as embedding area of VN rather than using traditional graph partition. Then the algorithm extracts available substructures within some distance constraint as candidates. Finally, the optimal candidate is found by comparison of graph entropies of all candidates.

Experimental results on the Alevin Platform have shown that our algorithm exhibits some merits regarding a group of principal VNE evaluation metrics. It was also observed that there is no dominance of our algorithm on some of these metrics. Future research has been scheduled to explore improvements to this problem.

## Figures and Tables

**Table 1:** Parameters values used in GE-VNE.

Parameters	Values	Description
*r_k_*(*H*)	0	Radius of *S_k_*(*v*, *G*) for VN
*r_k_*(*G*)	20	Radius of *S_k_*(*v*, *G*) for SN
*r_j_* − *r_j_*_−1_	10	The increment of radius for *S_k_*(*v*, *G*)
*α*	3	Constant in Formula (13)
*c_i_* (1 ≤ *i* ≤ 7)	1–7	Weights in Formula (13)
*p*	6	Number of candidate areas
*s*	1/3	Number of candidate nodes
*w_i_* (1 ≤ *i* ≤ 3)	1	Initial distance factor in Formula (9)

**Table 2:** Algorithmic parameters.

Parameters	Description	Values
*dist*	Distance for candidates	20
*wCPU*	CPU weight	1
*wBw*	Bandwidth weight	1
*nodeoverload*	Node overload concerned	false
type	Type of routing	0
k	Number of *k*-shortest path	5

**Table 3:** Representative VNE approaches chosen for evaluation, the citation times updated on 8 July 2017.

Algorithm	Reference	Brief Description
DViNE-SP DViNE-PS	Chowdhury et al. [2]	VNE with coordinated strategy in two stages where node mapping is implemented by mixed integer programming (MIP) and link mapping with *k*-shortest paths. Google Scholar [24] citations: 415
GAR-SP	Yu et al. [5]	VNE preferentially using available resources for node mapping and *k*-shortest paths for link mapping. Google Scholar [24] citations: 998
RW-MM-SP RW-MM-PS	Cheng et al. [7]	VNE ranking nodes with topology properties for node mapping and *k*-shortest paths for link mapping. Google Scholar [24] citations: 346

**Table 4:** Descriptions of VNE evaluation metrics.

Metrics	Interpretations
ALS	The average of the proportion of occupied bandwidth on each link
AR	The ratio between the number of accepted VNRs and the total number of VNRs
CRR	The ratio of embedding cost and revenue
cost	The sum of the substrate resources allocated to the VNR
LU	The proportion of occupied bandwidth
LCPV	The link cost for embedding each VN

**Table 5:** Comparison of average results from executing algorithms 20 times on 7 × 7 Mesh.

Algorithms	AR	Cost	CRR	LCPV	LU	ALS
RW-MM-SP	84%	10.50	2.70	163.80	10.5	0.37
RW-MM-PS	41%	750.7	1.58	44.80	26.8	0.04
DViNE-SP	89%	116.0	2.07	122.75	14.2	0.29
DViNE-PS	66%	642.7	1.24	34.84	27.5	0.063
GE-VNE	86%	203.8	1.55	68.70	28.7	0.18
GAR-SP	74%	402.8	1.82	91.71	24.9	0.16

## Abbreviations

The following abbreviations are used in this manuscript:

InP	Infrastructure Provider
ISP	Internet Service Provider
SN	Substrate Network
VN	Virtual Network
VNE	Virtual Network Embedding
VNP	Virtual Network Provider
VNR	Virtual Network Request
